# Knowledge–practice gaps and dental service utilisation under India’s national oral health programme: a community-based study in Rural Odisha

**DOI:** 10.1186/s12903-026-08783-9

**Published:** 2026-06-05

**Authors:** Gunjan Kumar, Payal Dash, Delfin Lovelina Francis, Saravanan Sampoornam Pape Reddy, Shaswata Karmakar

**Affiliations:** 1 Department of Public Health Dentistry, Kalinga Institute of Dental Sciences (KIDS), Kalinga Institute of Industrial Technology (KIIT) Deemed to Be University, Bhubaneswar, India; 2 Department of Public Health Dentistry, Saveetha Dental College & Hospitals, Saveetha University, SIMATS, Chennai, India; 3 Department of Periodontology, Army Dental Corps, New Delhi, 110010 India; 4 Department of Periodontology, Manipal College of Dental Sciences, Manipal Academy of Higher Education, Manipal, India

**Keywords:** Oral Health, Health Knowledge, Attitudes, Practice, Rural Population, National Health Programs, Health Services Accessibility, Health Services Utilization, Oral Health Inequality

## Abstract

**Background:**

Despite National Oral Health Programme launched in India significant knowledge-practice gaps persist in rural populations. This study evaluates oral health literacy, behavioural determinants, and clinical outcomes among rural Odisha residents using contemporary health behaviour frameworks. However, evidence on behavioural pathways and public dental service utilisation under NOHP in rural populations remains limited.

**Methods:**

A community based cross-sectional study was conducted among 417 adults (≥ 18 years) through two-stage stratified random sampling in Satyabadi Block, Puri District (May-September 2024). Data was collected using a validated 20-item questionnaire assessing knowledge, attitudes and practices (KAP) regarding National Oral Health Programme (NOHP) and WHO 2013 for oral health examination. Statistical analyses included descriptive statistics, regression models, and structural equation modelling (SEM) to examine complex pathways linking knowledge, attitudes, practices, and clinical outcomes.

**Results:**

Oral health program knowledge was low (6.7%), while attitudes were favourable (75.5–84.7%). Despite this, service utilisation remained minimal (2.4%). Mean DMFT was 3.69 ± 1.80. SEM showed significant pathways from knowledge to practice (β = 0.458, *p* < 0.001), explaining 34.2% of variance in behaviour. A substantial knowledge–practice gap (95.2%) was observed.

**Conclusions:**

This study reveals a critical “know-do” gap where favourable attitudes fail to translate into preventive behaviours or service utilization. Structural barriers like geographic isolation, financial constraints, poor infrastructure may play a dominant role in individual-level factors. Multi-level interventions addressing systemic barriers, community health worker training, mobile dental units and behaviour change communication are imperative for NOHP effectiveness. Findings suggest that utilisation is likely influenced by structural barriers such as accessibility and service availability.

**Supplementary Information:**

The online version contains supplementary material available at https://doi.org/10.1186/s12903-026-08783-9.

## Introduction

Global Burden of Disease Study in 2017 estimated oral diseases as the most prevalent among 291 diseases which affects 3.58 billion people globally [1, 2]. In the year 2002-03, the National Oral Health Survey documented a high prevalence of 50–90% dental caries, 85–90% periodontal diseases and oral cancer accounting for 30% of all cancers nationally in India [3, 4]. More recent evidence continues to highlight persistent oral health inequalities and limited service utilisation in rural India, with national estimates indicating that less than one-quarter of the population utilizes dental services [5]. Also, the study showed that the rural population experience 1.8–2.3 times higher disease prevalence compared to the urban counterparts [5, 6].

To overcome the oral health disease burden, the National Oral Health Programme (NOHP) was launched in India during the 12th Five-Year Plan (2014-15) under the National Health Mission [7]. NOHP highlights a major shift toward preventive, promotive, community-oriented strategies underscoring equity and integration with primary healthcare [8]. The strategic framework integrates dental care units at district hospitals and Community Health Centers, basic oral health services at Primary Health Centers, school-based oral health education, community awareness campaigns, healthcare worker capacity building and referral linkages [9, 10].

The study is grounded in established behavioural frameworks. The Knowledge–Attitude–Practice (KAP) model informed the assessment of behavioural domains, assuming that knowledge influences attitudes, which in turn shape practices [11, 12]. The Health Belief Model provided a conceptual basis for understanding perceived barriers and service utilisation behaviour in resource-constrained settings [13]. Structural Equation Modelling (SEM) was employed to operationalize these theoretical relationships by examining direct and indirect pathways between sociodemographic factors, knowledge, attitudes, practices, and clinical outcomes [14, 15].

A substantial proportion of the rural population in India faces multidimensional oral health access disparities. The factors being, geographic barriers; 15–25 km distances to dental facilities with poor transportation. Although the World Health Organization cites an approximate benchmark of one dentist per 7,500 population for adequate service coverage, India’s historical dentist-to-population ratio has been reported at approximately 1:30,000, with a substantial urban concentration of dental professionals limiting rural service availability [16, 17]. Economic and cultural factors further exacerbated the challenges. Evidence from literature on out-of-pocket dental expenditure averages 95–98% in rural India, with catastrophic health expenditure (> 10% annual household income) affecting 18–22% of families, poor oral health priority, disease normalization, traditional healer preference, gender disparities further compound the situation [18–20].

Odisha is classified as an Empowered Action Group (EAG) state by the Government of India, a designation used for states facing comparatively greater developmental and health system challenges. A ‘block’ refers to an administrative sub-district unit, while ‘gram panchayats’ are village-level local self-governance bodies. The state exhibits high disparities with 83.3% rural population, 42.5% poverty and inadequate healthcare infrastructure [21]. Satyabadi Block in Puri District of Odisha was selected as a representative rural administrative unit characterized by limited dental infrastructure, dependence on primary healthcare services, and socio-economic constraints typical of underserved regions in eastern India. This setting provides an appropriate context to evaluate the real-world implementation of the National Oral Health Programme in a resource-limited environment.

Despite the implementation of the National Oral Health Programme in India, significant knowledge–practice gaps persist, there is limited evidence on how behavioural determinants translate into service utilisation and clinical outcomes in rural populations [22]. Existing studies have largely focused on awareness or isolated components of oral health, with minimal exploration of integrated behavioural pathways using advanced analytical approaches such as structural equation modelling (SEM). Therefore, this study aimed to (1) assess oral health knowledge, attitudes, practices, and clinical status among adults in a rural population, and (2) examine the behavioural pathways linking these factors to service utilisation and oral health outcomes using structural equation modelling.

## Methodology

### Study design and setting

This community based cross-sectional analytical study was conducted in Satyabadi Block, Puri District, Odisha (May–September 2024), which comprises 22 gram panchayats (village-level local self-government administrative units) and is served by one Community Health Centre (with limited dental facilities), six Primary Health Centres (without dedicated dental personnel), and a small number of private practitioners. This study is reported in accordance with the Strengthening the Reporting of Observational Studies in Epidemiology (STROBE) guidelines for cross-sectional studies [23].

#### Official permission

Ethical Clearance from KIIT Institutional Ethics Committee (KIIT/KIMS/IEC/1477/2023) and administrative permissions were obtained. Informed consent was obtained from all the participants prior to enrolment. For participants who were unable to read or write, the study information was explained verbally in their local language (Odia), and informed consent was obtained using a thumb impression in the presence of an impartial witness, in accordance with institutional ethical guidelines and the Declaration of Helsinki [24].

### Sample size and sampling

**Fig. 1:**
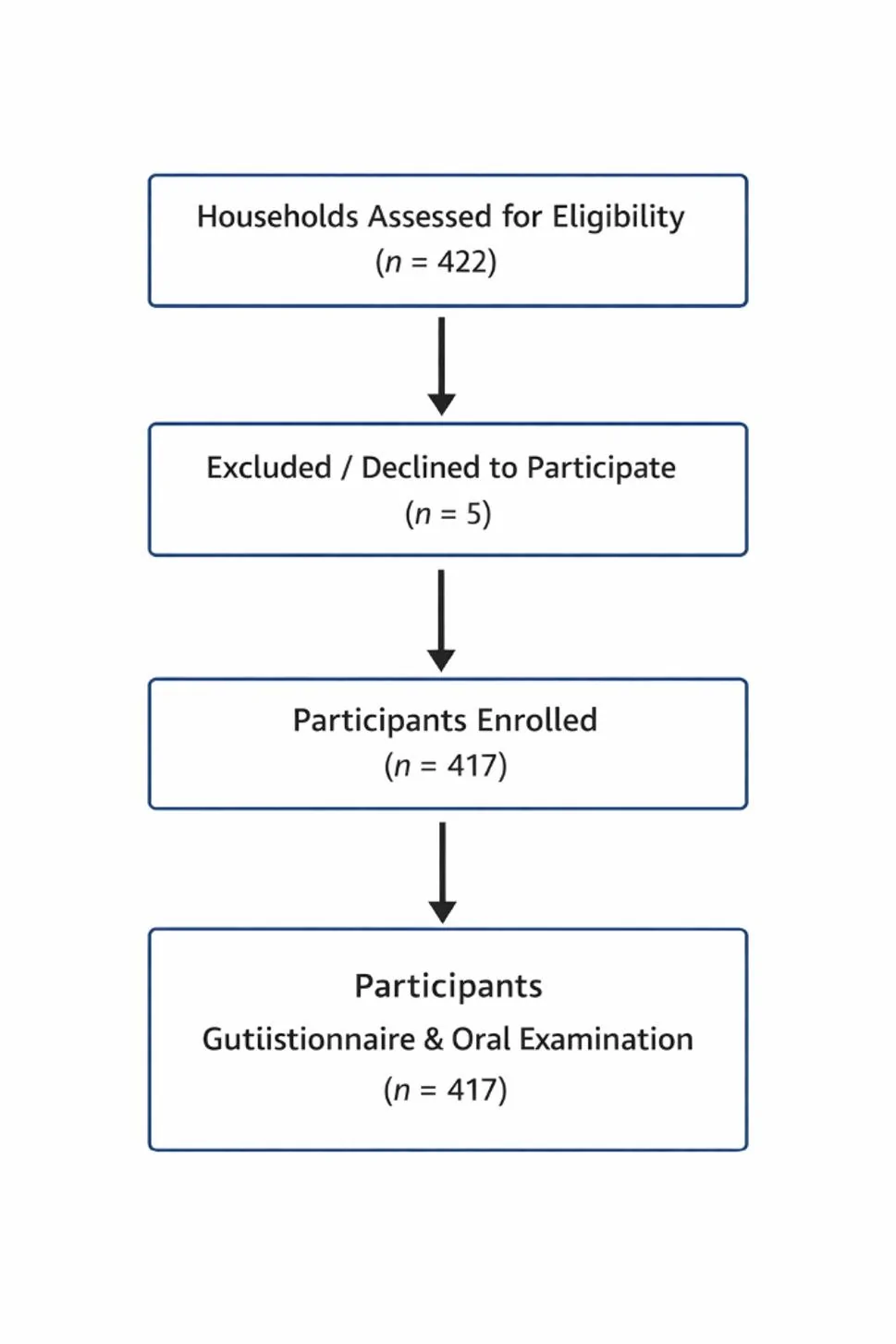
STROBE flowchart

Stratified random sampling was used for recruitment, with gram panchayats serving as strata to ensure proportional geographic representation. Within each selected household, one eligible participant was chosen using a simple random method (lottery method) to minimize selection bias. Sample size was calculated using Cochran’s formula [25] with finite population correction. Assuming a prevalence of 60% based on previous Indian epidemiological studies [6], 95% confidence level (Z = 1.96), precision of 5%, and design effect of 1.5, the minimum required sample size was 384. After accounting for 10% non-response, a final sample size of 422 was targeted. Yielding a response rate of 98.8%, 417 participants completed all assessments in total (Fig. 1- STROBE Flowchart).

#### Inclusion and exclusion criteria

Participants present during the time of data collection and aged 18 years and above and those who gave consent were included in the study. Individuals were excluded if they were temporary visitors or non-residents of the selected villages; unable to provide informed consent (including those with cognitive impairment or intellectual disability without a legally acceptable representative); had communication barriers that precluded completion of the questionnaire even with assistance; or were medically unstable or acutely ill at the time of interview or clinical examination.

### Data collection instruments

#### KAP questionnaire development

Comprehensive 20-item instrument (Supplement file S1) developed through expert consultation and the content was validated.

1. Sociodemographic (7 items): Age, gender, education, occupation, income, marital status, social category. Modified Kuppuswamy Socioeconomic Scale (2022) assessed socioeconomic status [26].
2. Knowledge (5 items): NOHP awareness, service knowledge, camp awareness, disease prevention understanding, education session exposure. Scoring: 0–5 (Poor: 0–1, Fair: 2–3, Good: 4–5).
3. Attitude (7 items): Program implementation necessity, service willingness, recommendation intention, disease burden beliefs, financial burden beliefs, infrastructure support, sensitization approval. Scoring: 0–7 (Negative: 0–3, Neutral: 4, Positive: 5–7).
4. Practice (8 items): Primary Health Centre (PHC)/ Community Health Centre (CHC) utilization, referral follow-up, treatment history, tobacco use, cessation attempts, counselling receipt. Scoring: 0–8 (Poor: 0–2, Fair: 3–5, Good: 6–8).

Score categorization was based on distributional thresholds and prior KAP studies in similar settings [27], where higher scores indicate better knowledge, attitude, and practice levels. Self-reported practices may be subject to social desirability bias; this was minimized through anonymous data collection and standardized interviewer training.

Validation: The draft questionnaire was reviewed by a panel of subject experts in public health dentistry, behavioural science, and community health to assess relevance, clarity, and domain representation. The Content Validity Index (CVI) was calculated (overall CVI = 0.89), and items requiring modification were revised accordingly. The instrument was translated into Odia using forward translation and independently back-translated to ensure semantic equivalence. A pilot study was conducted among 30 adults in a comparable rural village to assess comprehensibility and feasibility; minor wording adjustments were made based on feedback. Internal consistency reliability was evaluated using Cronbach’s alpha (knowledge = 0.82; attitude = 0.87; practice = 0.79). Test–retest reliability was assessed by re-administering the questionnaire to the pilot sample after a short interval, and intraclass correlation coefficients (ICC) demonstrated strong stability (knowledge = 0.88; attitude = 0.91; practice = 0.85). Construct validity was not formally assessed using factor analysis but was consistent with previously validated KAP instruments in similar settings. However, content validity and internal consistency measures were applied to ensure reliability.

### Clinical examination

Clinical oral examinations were conducted using the WHO Oral Health Assessment Form (2013) [28]. Dental caries experience was assessed using the Decayed, Missing (due to caries), and Filled Teeth (DMFT) index, with scores ranging from 0 to 32 and severity categorized according to WHO criteria as very low (0.0–1.1), low (1.2–2.6), moderate (2.7–4.4), high (4.5–6.5), and very high (> 6.6). Periodontal status was evaluated using the Community Periodontal Index (CPI) by examining six index teeth, with scores recorded as 0 (healthy), 1 (bleeding on probing), 2 (presence of calculus), 3 (periodontal pockets of 4–5 mm), and 4 (periodontal pockets ≥ 6 mm). Clinical examinations were conducted by a single calibrated examiner over a four-month data collection period (May–September 2024). On average, 10–15 participants were examined per field visit day. The examiner was a trained public health dentist experienced in community oral health surveys. Calibration exercises were performed prior to the study to ensure diagnostic consistency. Intra-examiner reliability was assessed by re-examining 10% of the study participants (*n* = 40) after a short interval, and agreement was evaluated using Cohen’s kappa statistics, yielding values of 0.912 for DMFT and 0.887 for CPI, indicating diagnostic consistency.

### Statistical analysis

Statistical analysis was conducted using Microsoft Excel 2019, IBM SPSS Statistics version 27.0, and SmartPLS version 4.0, with a two-sided significance level of 0.05. Descriptive statistics were summarized using means, standard deviations, and 95% confidence intervals. A design effect (DEFF = 1.5) was incorporated in the sample size calculation to account for clustering at the gram panchayat level. Clustering was not explicitly modelled analytically due to the cross-sectional design and single-level outcome structure in regression analyses as one participant was selected per household, minimizing intra-household correlation.

Bivariate associations were examined using chi-square or Fisher’s exact tests for categorical variables and independent *t*-tests or one-way analysis of variance (ANOVA) for continuous variables, with Tukey or Games–Howell post-hoc comparisons as appropriate. Associations between behavioural measures were assessed using Spearman’s rank correlation coefficients, as the variables were not normally distributed based on normality testing.

Multivariable analyses included multiple linear regression models to identify predictors of DMFT and CPI scores. Model assumptions were evaluated using standard diagnostic procedures, including assessment of residual distributions, homoscedasticity, multicollinearity (variance inflation factor < 5), and influential observations. Binary logistic regression models were used to identify determinants of adequate knowledge, positive attitudes, and favourable practices, with variables selected based on bivariate screening and results expressed as adjusted odds ratios with 95% confidence intervals.

Exploratory behavioural pathway analysis was performed using partial least squares structural equation modelling (PLS-SEM) to examine relationships between sociodemographic factors, knowledge, attitudes, practices, and clinical outcomes. PLS - SEM was chosen due to its suitability for exploratory modelling, ability to handle non-normal data, and robustness with relatively small sample sizes. Measurement model adequacy was assessed using indicator loadings, composite reliability, average variance extracted, and heterotrait–monotrait ratios, while structural model performance was evaluated using coefficients of determination (R²) and bootstrapped path estimates based on 5,000 resamples. Practice shortfall was quantified as the percentage difference between maximum possible and observed practice scores, with stratified comparisons and effect sizes estimated using Cohen’s *d*. Direct paths from sociodemographic variables to practice were not specified in the final model because preliminary model testing indicated that their effects were primarily mediated through knowledge and attitudes. Given the exploratory nature of the study, no formal correction for multiple comparisons was applied. Knowledge–practice gap was calculated as: [(maximum possible practice score − observed mean practice score) / maximum possible score] × 100.

## Results

### Sociodemographic characteristics

A total of 417 adults participated out of 422 eligible individuals approached (response rate: 98.8%). Most participants were aged 18–45 years (*n* = 293, 70.3%), 254 (60.9%) were male. The observed demographic distribution reflects the underlying rural population structure of the study area, characterized by a predominance of younger and economically active individuals, which is consistent with census patterns in similar rural settings. 141 (33.8%) had only primary education and the majority were married (*n* = 378, 90.6%) and engaged in unskilled or semi-skilled occupations. Detailed sociodemographic characteristics are presented in Supplementary File S2 for category definitions).

### Knowledge, attitude, and practice

**Fig. 2:**
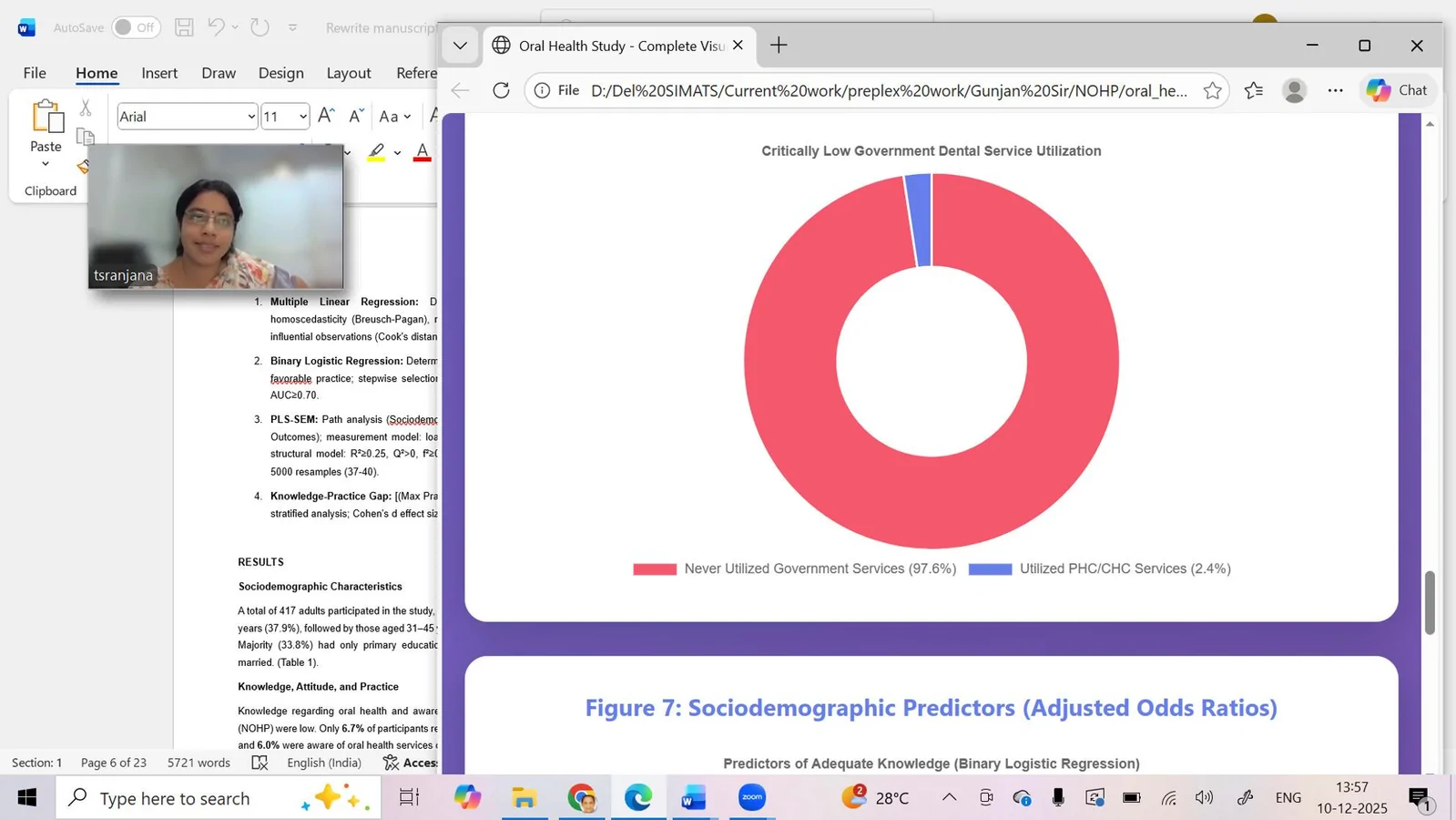
Distribution of oral health service utilisation among study participants

Awareness of oral health programmes was limited. Only 28 participants (6.7%) had heard of any oral health programme, and 25 (6.0%) were aware of oral health services under government initiatives. Knowledge of oral disease prevention was reported by 41 participants (9.8%). In contrast, attitudes toward oral health services were very favourable (*n* = 315, 75.5%). 315 participants (75.5%) supported government oral health programme implementation, and 353 participants (84.7%) expressed willingness to use services if available. Despite positive attitudes, oral health practices were poor. Most participants (*n* = 407, 97.6%) had never visited a PHC or CHC for dental care, and only 8 (1.9%) reported recent dental service utilization (past 12 months). Practice indicators primarily reflected healthcare utilisation behaviour, which was considered a proxy for applied oral health behaviour in the context of public service delivery. Tobacco use was common(*n* = 395, 94.7%), while cessation attempts were infrequent (*n* = 16, 3.8%). Health service utilization patterns are shown in Fig. 2. A substantial knowledge–practice gap of 95.2% (95% CI: 93.8–96.6%) was observed based on the defined scoring method. Detailed tables are presented in Supplementary File S2.

### Clinical oral health status

The overall mean DMFT score was 3.69 ± 1.80, corresponding to a moderate level of caries experience (which falls within the moderate caries severity category according to World Health Organization criteria for adult populations). The mean decayed component was 1.62 ± 0.99, and the mean missing component due to caries was 1.03 ± 1.16, together accounting for the majority of the DMFT score, while filled teeth were uncommon. DMFT scores did not differ significantly across age groups, (see Table 1 for component definitions and statistical comparisons). The median DT score was 2 (IQR: 1–2), MT score was 1 (IQR: 0–1), FT score was 0 (IQR: 0–0), and total DMFT score was 4 (IQR: 3–5) across all age groups. Kruskal–Wallis analysis revealed no statistically significant difference across age groups (*p* > 0.05), with minimal effect size, indicating negligible practical difference. This finding is consistent with moderate caries experience reported in national surveys and comparable regional studies.

**Table 1:** DMFT index scores by age group (*N* = 417)

Age Group	DT (Mean ± SD)	MT due to Caries (Mean ± SD)	MT due to Other Reasons (Mean ± SD)	FT (Mean ± SD)	DMFT (Mean ± SD)	DMFT Median (IQR)
18–30	1.60 ± 0.96	0.93 ± 1.04	0.01 ± 0.08	1.12 ± 0.86	3.66 ± 1.74	4 (3–5)
31–45	1.69 ± 0.95	1.16 ± 1.28	0.03 ± 0.17	0.95 ± 0.83	3.83 ± 1.85	4 (3–5)
46–60	1.47 ± 1.04	1.08 ± 1.24	0.01 ± 0.11	0.99 ± 0.80	3.55 ± 1.88	4 (3–5)
61 and above	1.72 ± 1.15	0.89 ± 1.04	0.00 ± 0.00	1.07 ± 0.71	3.67 ± 1.76	4 (3–5)
**Total**	**1.62 ± 0.99**	**1.03 ± 1.16**	**0.01 ± 0.12**	**1.03 ± 0.83**	**3.70 ± 1.80**	4 (3–5)
**F value**	0.941	1.239	1.206	1.168	0.439	
**p value**	0.421	0.295	0.307	0.321	0.725	

DMFT: Decayed, Missing, and Filled Teeth. Values are presented as mean ± standard deviation (SD). DT = Decayed Teeth; MT = Missing Teeth; FT = Filled Teeth; DMFT = Decayed, Missing, and Filled Teeth index. MT due to caries includes teeth extracted because of dental caries; MT due to other reasons includes extractions unrelated to caries. Comparisons across age groups were performed using one-way analysis of variance (ANOVA). p-values < 0.05 were considered statistically significant

**Fig. 3:**
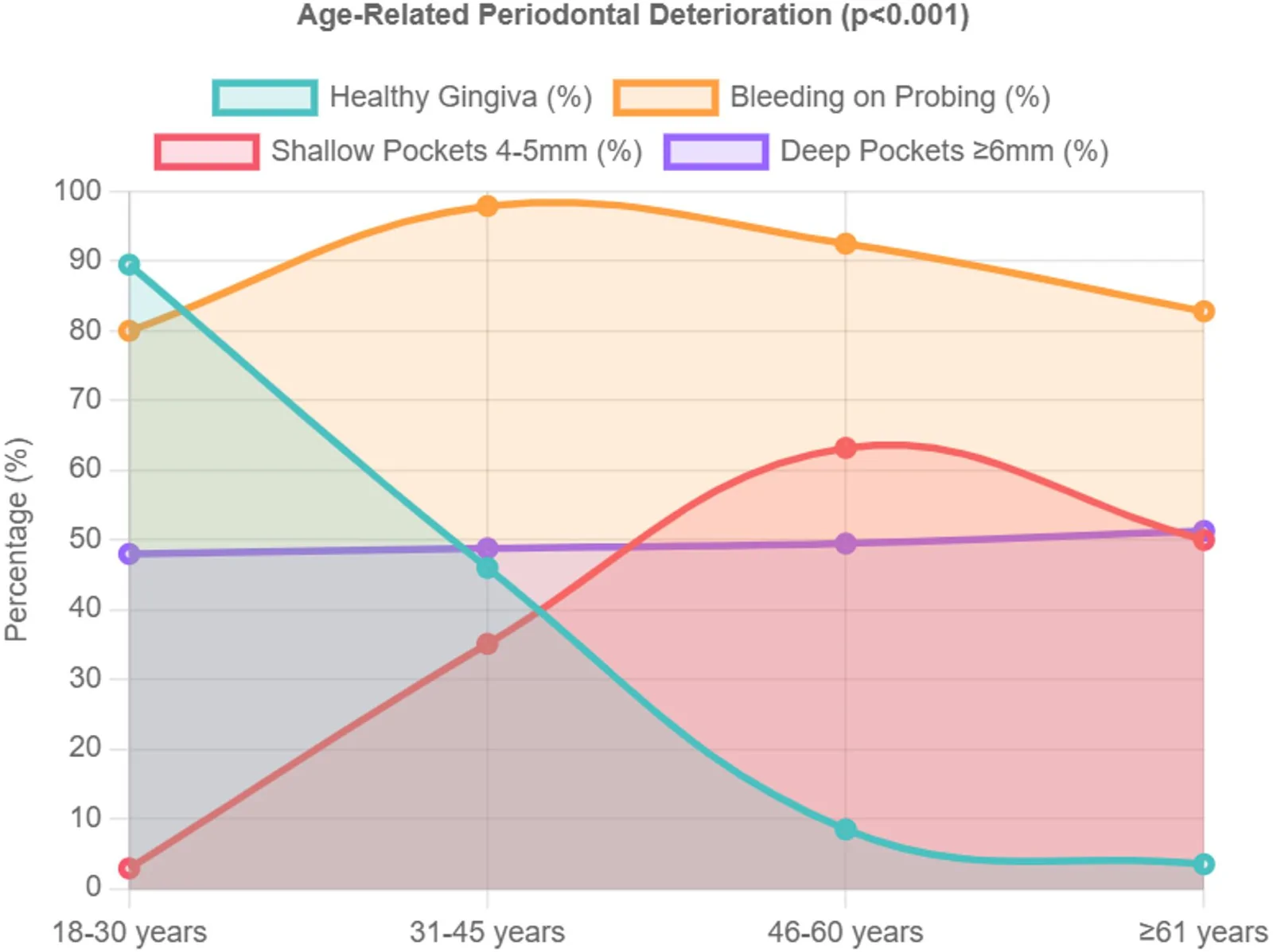
Distribution of Community Periodontal Index (CPI) scores across age groups. shows CPI score progression (line graph) across age categories, with error bars indicating standard deviations

Periodontal status showed significant age-related variation. The proportion of healthy sextants decreased significantly with age (*p* < 0.001). Bleeding (*p* = 0.001), periodontal pockets ≥ 4 mm (*p* < 0.001), and ≥ 6 mm (*p* = 0.001) also showed statistically significant differences across age groups. However, calculus did not differ significantly by age (*p* = 0.121). The high prevalence of deep periodontal pockets (≥ 6 mm) indicates advanced periodontal disease and a potential need for complex periodontal intervention. Participants aged ≥ 61 years had a higher mean number of sextants with periodontal pockets ≥ 4 mm (1.50 ± 0.50) and ≥ 6 mm (1.51 ± 0.06) compared to younger age groups (*p* < 0.001). CPI distributions by age group are presented in Table 2 (CPI scoring criteria) and Fig. 3 (graphical interpretation). Age-stratified analysis demonstrated a progressive deterioration in periodontal status with increasing age, consistent with the cumulative nature of periodontal disease.

**Table 2:** Community periodontal index scores by age group (*N* = 417)

Age Group	Healthy (Mean ± SD)	Bleeding (Mean ± SD)	Calculus (Mean ± SD)	Pocket 4 mm (Mean ± SD)	Pocket 6 mm (Mean ± SD)
18–30	0.90 ± 0.31	0.80 ± 0.49	0.78 ± 0.42	0.03 ± 0.17	0.48 ± 0.05
31–45	0.46 ± 0.50	0.98 ± 0.34	1.78 ± 0.42	0.35 ± 0.48	0.49 ± 0.04
46–60	0.08 ± 0.28	0.92 ± 0.27	0.88 ± 0.33	1.63 ± 0.48	1.50 ± 0.05
61 and above	0.03 ± 0.18	0.83 ± 0.38	0.86 ± 0.35	1.50 ± 0.50	1.51 ± 0.06
**Total**	**0.41 ± 0.49**	**0.90 ± 0.38**	**0.82 ± 0.39**	**0.36 ± 0.48**	**0.50 ± 0.02**
**F value**	107.804	5.655	1.948	37.282	6.941
**p value**	0.000*	0.001*	0.121	0.001*	0.001*

Values are presented as mean ± standard deviation (SD). CPI = Community Periodontal Index, assessed according to WHO Oral Health Surveys: Basic Methods (5th edition). Healthy = CPI score 0; Bleeding = CPI score 1; Calculus = CPI score 2; Pocket 4 mm = CPI score 3; Pocket 6 mm = CPI score 4. Comparisons across age groups were performed using one-way analysis of variance (ANOVA) to evaluate age-related differences in periodontal status. *p < 0.05 indicates statistical significance

### Associations between behavioural measures and clinical outcomes

Attitude scores were weakly correlated with practice scores (*r* = 0.193, *p* < 0.05). Knowledge and practice scores showed a moderate correlation (see Table 3 correlation method and significance levels). Although statistically significant, the observed correlations were weak (ρ < 0.3), suggesting limited clinical relevance. However, none of the behavioural measures were significantly associated with DMFT scores in multivariable linear regression (see Table 4 for regression model parameters and variable definitions). Multiple linear regression analysis was performed with DMFT score as the dependent variable and knowledge (KTOTAL), attitude (ATOTAL), and practice (PTOTAL) scores as independent predictors. None of the predictors showed a statistically significant association with DMFT (all *p* > 0.05) representing an important negative finding. The model demonstrated very low explanatory power (adjusted R² = 0.009), suggesting that behavioural variables alone do not adequately explain variation in cumulative caries experience.

**Table 3:** Correlation matrix - knowledge, attitude, and practice scores

Variable	Knowledge	Attitude	Practice
**Knowledge**	1.000	0.193**	0.535**
**Attitude**	0.193**	1.000	-0.049
**Practice**	0.535**	-0.049	1.000

Spearman’s rank correlation coefficient (ρ) was used to assess linear associations between variables. **Correlation is significant at the 0.01 level (two-tailed). *p* < 0.05 was considered statistically significant. Higher scores indicate better knowledge, attitude, and practice

**Table 4:** Multiple linear regression analysis showing the association between knowledge, attitude, and practice scores and DMFT

Variable	Unstandardized Coefficient (B)	Std. Error	Standardised Coefficient(β)	t	Sig.	95% CI Lower	95% CI Upper
(Constant)	4.654	1.163	—	4.003	0.000*	2.350	6.959
KTOTAL	-0.020	0.131	-0.015	-0.153	0.879	-0.280	0.240
ATOTAL	-0.081	0.123	-0.063	-0.659	0.511	-0.324	0.162
PTOTAL	-0.111	0.101	-0.107	-1.101	0.273	-0.312	0.089

B = Unstandardized regression coefficient; β = Standardized regression coefficient; CI = Confidence Interval. KTOTAL = Total knowledge score; ATOTAL = Total attitude score; PTOTAL = Total practice score. Multiple linear regression analysis was performed with DMFT as the dependent variable. 95% CI indicates the confidence interval for the unstandardized coefficient. *p < 0.05 indicates statistical significance

### Behavioural pathway analysis

**Fig. 4:**
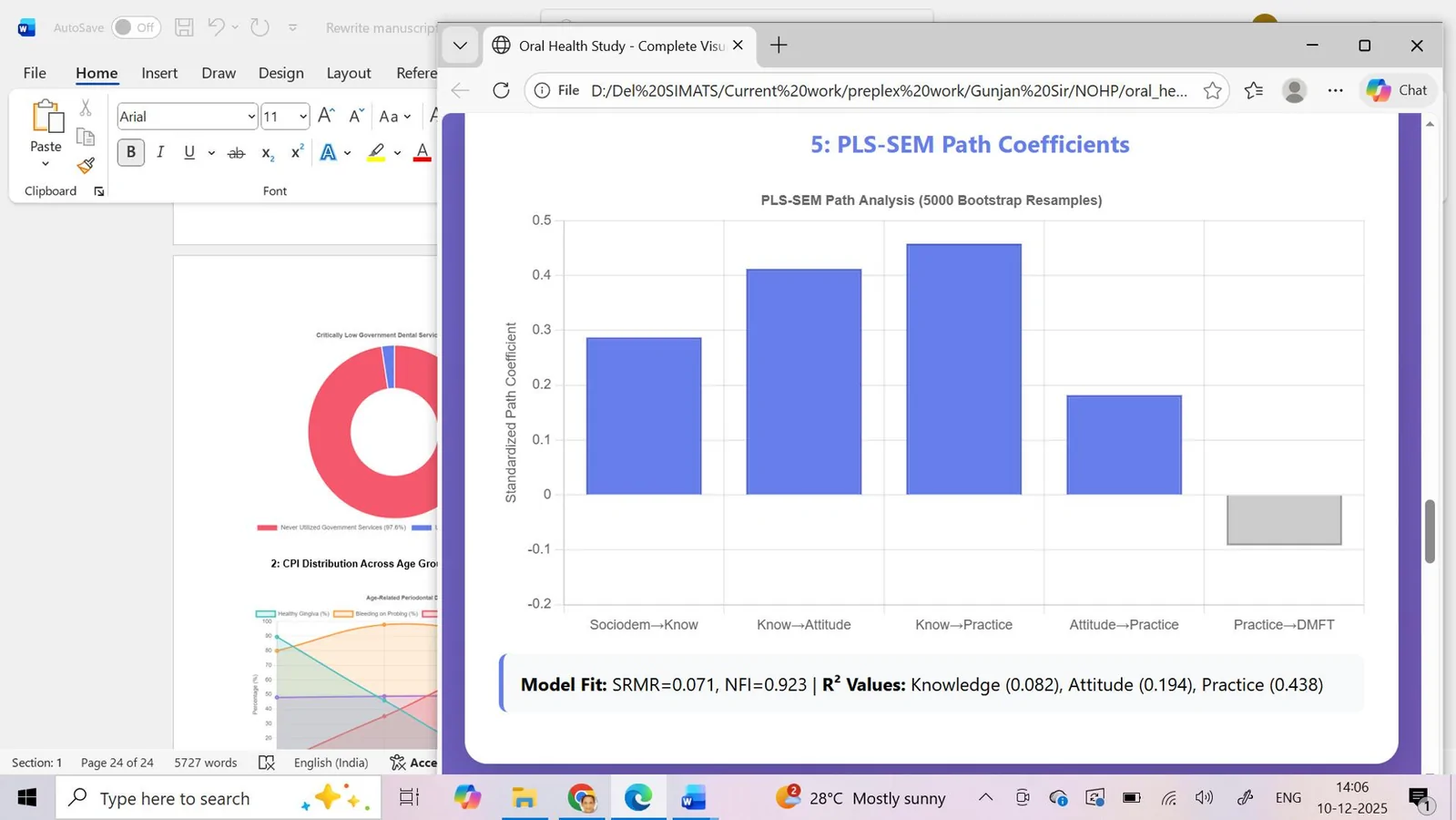
Structural equation model illustrating relationships between sociodemographic factors, knowledge, attitudes, practices, and oral health outcomes. Model Fit: SRMR=0.071, NFI=0.923 | R² Values: Knowledge (0.082), Attitude (0.194), Practice (0.458). Knowledge exerts strongest direct effect on practice (β=0.458), with additional indirect effect through attitude. Attitude partially mediates knowledge-practice relationship. Sociodemographic factors influence practices both directly and indirectly through knowledge/attitude. However, KAP dimensions collectively explain minimal DMFT variance, suggesting clinical outcomes depend primarily on long-term accumulated exposures, biological factors, and structural determinants rather than recent knowledge/behavioral changes

Exploratory partial least squares structural equation modelling indicated that sociodemographic factors were associated with oral health knowledge and attitudes. Specifically, knowledge demonstrated a strong positive association with practice (β = 0.458, *p* < 0.001), and attitude showed a smaller but statistically significant association with practice (β = 0.182, *p* = 0.042). The pathway between practice and DMFT was not statistically significant (*p* > 0.05) (see Table 5 for model fit indices, path coefficients, and effect sizes; and Fig. 4 for structural model pathways and fit indices). SEM analysis demonstrated that knowledge significantly influenced attitudes and practices, indicating a behavioural pathway; however, these pathways did not translate into meaningful effects on clinical outcomes.

**Table 5:** PLS-SEM path analysis results

Path	Direct Effect (β)	95% CI	t-value	*p*-value	f²
Sociodemographic → Knowledge	0.287	0.168–0.401	4.683	< 0.001	0.090
Sociodemographic → Attitude	0.154	0.042–0.269	2.691	0.007	0.026
Knowledge → Attitude	0.412	0.298–0.521	7.214	< 0.001	0.205
Knowledge → Practice	0.458	0.342–0.569	7.892	< 0.001	0.312
Attitude → Practice	0.182	0.069–0.295	3.176	0.002	0.049
Practice → DMFT	-0.092	-0.211-0.024	-1.583	0.114	0.009

β = standardized path coefficient; CI = bias-corrected 95% confidence interval (bootstrapped); f² = effect size; Model fit indices: SRMR = 0.071; NFI = 0.923; R²: Knowledge (0.082), Attitude (0.194), Practice (0.438), DMFT (0.009); Q²: Knowledge (0.041), Attitude (0.098), Practice (0.221), DMFT (− 0.002); p-values derived from bootstrapping in PLS-SEM, with *p* < 0.05 considered statistically significant. (SRMR = Standardized Root Mean Square Residual; NFI = Normed Fit Index; R² = Coefficient of determination indicating explained variance of endogenous constructs; Q² = Predictive relevance assessed using blindfolding procedure)

## Discussion

This study provides comprehensive evidence of a substantial disconnect between oral health knowledge, attitudes, and service utilization among adults in rural Odisha, within the operational framework of National Oral Health Programme (NOHP) in India. Despite nearly a decade since NOHP implementation, awareness of programme components and utilization of public dental services remained critically low, even though attitudes toward government-supported oral healthcare were overwhelmingly positive. These findings underscore a persistent “knowledge–practice gap” likely driven primarily by structural and systemic barriers rather than individual unwillingness or negative perceptions. Differences across regions may be attributed to variations in healthcare infrastructure, cultural practices, and accessibility of public dental services, highlighting the context-specific nature of oral health behaviour.

### Knowledge–practice gap and programme visibility

Only a small proportion (6.7%) of participants were aware of NOHP or related oral health services, highlighting inadequate programme visibility and weak community-level dissemination. The results were substantially lower than Nayak et al.‘s 24% national health program awareness among adolescents indicating rural-urban disparities and inadequate community outreach [29]. Rural–urban disparities remain a critical determinant of oral health outcomes, driven by workforce maldistribution, infrastructure gaps, and limited integration of dental services within primary healthcare systems. These disparities contribute to delayed care-seeking and reliance on symptomatic treatment. Compared with other national public health initiatives in India, such as health insurance or maternal–child health programmes, oral health appears to receive lower policy prioritization and communication investment. However, this aligns with previous studies documenting 60–85% oral health knowledge deficits in rural Indian populations reporting comparably low oral health literacy, particularly in populations with limited formal education and restricted access to health information [30–33].

The observed 95% knowledge–practice gap, reflects a failure of translation from awareness or intent into action. Importantly, this gap does not appear to stem from attitudinal resistance. Also, most participants expressed willingness to utilize services and supported government-led oral health initiatives. This underscores that individual-level behavioural readiness alone is not sufficient to produce meaningful health-seeking behaviour in the absence of service enabling environments, which is widely described in health behaviour and ecological models [34]. This mirrors findings from Nagarjuna et al. in Andhra Pradesh, where fear (48.6%), time constraints (45.8%), and costs prevented service use despite positive attitudes [35].

### Structural barriers to service utilization

Poor utilization of dental services at Primary Health Centres (PHCs) and Community Health Centres (CHCs) explains the barrier to access. Factors like geographic distance, transportation costs, limited availability of dental specialist, clinic hours and money lost due to lost wages play a crucial role in service utilisation. These findings are consistent with prior research where accessibility, affordability and health system capacity were discussed as key determinants of dental care utilization in rural settings [36].

Within the NOHP framework, dental services at the primary care level are intended to function as the backbone of preventive and basic curative care. However, the present findings suggest that this intent has not translated into functional service delivery in remote areas. The discrepancy between programme design and ground-level implementation highlights the need for stronger integration of oral health into primary healthcare, supported by workforce strengthening, mobile dental units, and community-based outreach mechanisms [37, 38]. These findings suggest that structural barriers likely play a dominant role in shaping service utilisation patterns.

### Clinical disease burden and limited preventive care

The study population exhibited a moderate mean DMFT score with a high prevalence of untreated caries and missing teeth, alongside widespread periodontal disease with clear age-related deterioration, this aligns with national oral health survey findings and prior Indian epidemiological data. The predominance of missing and decayed components, coupled with minimal restorative treatment, reflects delayed care-seeking and reliance on extraction-based management. These patterns are characteristic of health systems where preventive and conservative dental services are either unavailable or underutilized [39].

Notably, behavioural indicators such as knowledge, attitudes, and reported practices showed minimal association with DMFT scores. This finding is consistent with the understanding that cumulative oral disease experience reflects long-term exposures, dietary patterns, socioeconomic conditions, and historical access to care rather than recent behavioural change. Thus, while behavioural interventions remain essential, their impact on clinical outcomes is likely to be realized only when embedded within accessible and continuous service delivery systems [40].

### Behavioural pathways and implications for intervention design

Exploratory pathway analysis demonstrated that knowledge and attitudes were strongly associated with oral health practices, indicating that behavioural constructs remain relevant determinants of service-seeking behaviour. However, the weak linkage between practices and clinical outcomes reinforces the need to move beyond individual-focused education models (Fig. 3). Interventions that address upstream determinants such as service availability, workforce distribution, and financial protection are likely to yield greater population-level impact.

These findings align with ecological and implementation science frameworks, which emphasize that health behaviours are shaped by multilevel influences, including policy, infrastructure, and community context. For NOHP to achieve its intended objectives, behavioural change communication must be coupled with tangible service delivery reforms that reduce access barriers and enhance trust in public dental facilities [41].

### Study limitations

This study has several limitations. The cross-sectional design precludes causal inference, particularly for structural equation modelling pathways, which should be interpreted as associative rather than causal. Behavioural measures were self-reported and may be subject to social desirability and recall bias, potentially leading to over-reporting of favourable practices. Selection bias may be present due to the inclusion of a single participant per household, despite random selection. The study setting in a single rural block limits generalizability to other regions with differing health system capacities. Measurement limitations include the partial recording nature of CPI, which may underestimate periodontal disease burden, and the use of composite KAP scores that may not fully capture behavioural complexity. Additionally, the study assessed dental service utilization within the past 12 months, and lifetime utilization patterns were not captured. Despite these limitations, the study provides valuable insights into systemic gaps affecting oral health service delivery in rural India.

### Policy and programmatic implications

The strong public support observed in this study represents an important opportunity for programme strengthening. These findings suggest that dental practitioners should prioritize community-based preventive approaches, outreach services, and early screening rather than relying solely on facility-based care. Strengthening the role of primary care dentists within the public health system, particularly at the Primary Health Center level, may improve early detection, referral pathways, and continuity of care. Strategies such as training community health workers in basic oral health promotion, deploying mobile dental clinics, integrating oral health screening into existing primary care services, and leveraging school- and community-based platforms could substantially improve reach and utilization. Aligning oral health messaging with broader non-communicable disease and primary healthcare initiatives may also enhance programme visibility and sustainability. Similar approaches have demonstrated success in other LMIC contexts and align with WHO’s integration and equity-driven oral health policy recommendations [42].

### Expanded policy and implementation implications

Beyond documenting gaps in knowledge, attitudes, and practices, the present findings have important implications for the design and implementation of oral health policy in rural and underserved settings. The consistently high willingness to utilize public dental services observed in this population indicates that demand-side resistance is not the primary constraint. Instead, the findings point to a supply-side and system-level failure, where service availability, accessibility, and continuity are insufficient to translate latent demand into utilization [43]. This distinction is critical for policy planning, as it suggests that further investment in awareness campaigns alone is unlikely to yield meaningful improvements unless accompanied by tangible service delivery reforms. From an implementation science perspective, these findings illustrate a classic “know–do gap”, in which intervention acceptability is high but feasibility, reach, and routine delivery are constrained by system-level barriers rather than individual resistance.

Within the NOHP framework, strengthening oral health integration into primary healthcare represents a pragmatic and scalable approach. Training frontline health workers, such as ASHAs and auxiliary nurse midwives, to deliver basic oral health education, screening, and referral support could substantially improve early detection and community trust. Evidence from other low- and middle-income country settings indicates that task-sharing models and community-based oral health promotion can improve service uptake when formal dental infrastructure is limited [44]. Also, mobile dental units and periodic outreach camps may serve as temporary solutions in geographically remote areas, particularly for preventive and basic curative care.

Also, high out-of-pocket expenditure remains a major limitation factor for dental care utilization in rural India. Oral health insurance schemes which are publicly funded or integrating basic dental procedures into existing primary care benefit packages could reduce financial barriers and improve equity. Incorporating oral health messaging within broader non-communicable disease and primary healthcare initiatives rather than delivered as standalone campaigns, thereby leveraging existing platforms and improving sustainability [45].

Finally, the minimal association between behavioural indicators and clinical outcomes observed in this study underscores the need for longitudinal monitoring and implementation research. Future evaluations of NOHP should move beyond coverage indicators to assess service quality, continuity of care, and clinical effectiveness over time. Such evidence is essential to inform adaptive policy responses and ensure that oral health programmes translate into measurable improvements in population oral health [46].

## Conclusion

In summary, this study highlights a substantial knowledge–practice gap and low utilisation of public dental services despite favourable attitudes in a rural population. These findings indicate associations rather than causal relationships, reflecting the limitations of cross-sectional data. The results suggest that utilisation patterns may be influenced by systemic factors such as access and service availability. These findings may inform policy by highlighting the need to strengthen primary oral healthcare and integrate dental services within primary health systems. Strengthening primary care dental services and community-based preventive strategies may help bridge the gap between oral health awareness and service utilisation in rural populations.

## Supplementary Information

Supplementary Material 1.

Supplementary Material 2.

Supplementary Material 3.

## Data Availability

Data available upon reasonable request to corresponding author.
