# Supplementary material for: Knowledge–practice gaps and dental service utilisation under India’s national oral health programme: a community-based study in Rural Odisha
Source: BMC Oral Health. 2026 Jun 5;26:1755. doi: 10.1186/s12903-026-08783-9 (PMC13584288; doi:10.1186/s12903-026-08783-9)
Supplement: Supplementary file 1 [file 12903_2026_8783_MOESM1_ESM.docx]

**Questionnaire**

**Section A: Socio-Demographic Details**

1. **Age (in years):**
   ☐ 18–30
   ☐ 31–45
   ☐ 46–60
   ☐ 61 and above
2. **Gender:**
   ☐ Male
   ☐ Female
3. **Educational status:**
   ☐ Illiterate
   ☐ Primary school
   ☐ Middle school
   ☐ High school
   ☐ Diploma
   ☐ Graduate
   ☐ Professional
4. **Occupation:**
   ☐ Professional (Doctor, Engineer, Lawyer, Manager, etc.)
   ☐ Semi-professional
   ☐ Semi-professional (High school teachers, Lecturers)
   ☐ Clerical / Shop owner / Farmer
   ☐ Skilled worker (Driver, Carpenter, Mechanic, etc.)
   ☐ Semi-skilled worker (Factory labourer, Car cleaner, etc.)
   ☐ Unskilled worker (Domestic servant, Peon, Watchman, etc.)
   ☐ Unemployed
5. **Monthly household income (₹):**
   ☐ ≤ 9,225
   ☐ 9,226 – 27,650
   ☐ 27,651 – 46,090
   ☐ 46,091 – 68,960
   ☐ 68,961 – 92,185
   ☐ 92,186 – 1,84,375
   ☐ ≥ 1,84,376
6. **Community:**
   ☐ General
   ☐ Other Backward Class (OBC)
   ☐ Scheduled Caste
7. **Marital status:**
   ☐ Married
   ☐ Single

**Section B: Knowledge Domain**

8. Have you heard about or come across any program related to oral health?
   ☐ Yes
   ☐ No
9. Do you know about the services available under any oral health program being provided in your village?
   ☐ Yes
   ☐ No
10. Do you know about any oral health–related camps being organised in your village?
    ☐ Yes
    ☐ No
11. Do you know about various oral diseases and their prevention?
    ☐ Yes
    ☐ No
12. Do you know about any oral health education session conducted in your village?
    ☐ Yes
    ☐ No

**Section C: Attitude Domain**

13. Do you think an oral health program ought to be implemented in the state?
    ☐ Yes
    ☐ No
14. Would you avail the dental services made available under the National Oral Health Programme (NOHP)?
    ☐ Yes
    ☐ No
15. Would you recommend your near or dear ones to avail dental services made available under NOHP?
    ☐ Yes
    ☐ No
16. Do you think NOHP will reduce the burden of oral health diseases?
    ☐ Yes
    ☐ No
17. Do you think NOHP will lessen the expenses on oral health treatment?
    ☐ Yes
    ☐ No
18. Do you think setting up Dental Care Units at district and sub-district levels is a good initiative under NOHP?
    ☐ Yes
    ☐ No
19. Do you think sensitization of the community about Basic Oral Health Care under NOHP is a welcome initiative?
    ☐ Yes
    ☐ No

**Section D: Practice Domain**

20. Have you ever visited the nearest PHC/CHC to avail dental health services?
    ☐ Yes
    ☐ No
21. Are you visiting the referral centre for follow-up dental visits?
    ☐ Yes
    ☐ No
22. Have you availed any dental treatment in the last one year?
    ☐ Yes
    ☐ No
23. Do you currently smoke any tobacco product such as bidis, cigarettes, cigars, or pipes?
    ☐ Yes
    ☐ No
24. Do you smoke tobacco products on a daily basis?
    ☐ Yes
    ☐ No
25. Were you young when you first started smoking?
    ☐ Yes
    ☐ No
26. During the past 12 months, have you ever tried to quit smoking?
    ☐ Yes
    ☐ No
27. During any visit to a doctor or other health worker in the past 12 months, were you counselled to quit smoking tobacco?
    ☐ Yes
    ☐ No
