# Supplementary material for: Knowledge–practice gaps and dental service utilisation under India’s national oral health programme: a community-based study in Rural Odisha
Source: BMC Oral Health. 2026 Jun 5;26:1755. doi: 10.1186/s12903-026-08783-9 (PMC13584288; doi:10.1186/s12903-026-08783-9)
Supplement: Supplementary file 3 [file 12903_2026_8783_MOESM3_ESM.docx]

**TABLES**

**Table 1: Sociodemographic Characteristics of Study Participants (N=417)**

| **Variables** | **Frequency** | **Percentage** |
| --- | --- | --- |
| **Age** |  |  |
| 18-30 | 158 | 37.9 |
| 31-45 | 135 | 32.4 |
| 46-60 | 78 | 18.7 |
| 61 and above | 46 | 11.0 |
| **Gender** |  |  |
| Male | 254 | 60.9 |
| Female | 163 | 39.1 |
| **Education** |  |  |
| Diploma | 41 | 9.8 |
| Graduate | 65 | 15.6 |
| High school | 88 | 21.1 |
| Illiterate | 70 | 16.8 |
| Middle school | 9 | 2.2 |
| Primary | 141 | 33.8 |
| Professional | 3 | .7 |
| **Occupation** |  |  |
| Clerical/Shop-owner/Farmer | 136 | 32.6 |
| Professional –  doctor, engineer, lawyer, manager etc | 7 | 1.7 |
| Semi-professional | 1 | .2 |
| Semi-professional –  high school teachers, lecturers | 7 | 1.7 |
| Semi-skilled worker-  factory labourer, car cleaner | 74 | 17.7 |
| Skilled worker - driver, carpenter, mechanic | 35 | 8.4 |
| Unemployed | 130 | 31.2 |
| Unskilled worker  (domestic servant, peon, watchman etc) | 27 | 6.5 |
| **Income** |  |  |
| ≤9225 | 116 | 27.9 |
| ≥1,84,376 | 11 | 2.6 |
| 27,651-46,090 | 71 | 17.0 |
| 46,091-68,960 | 31 | 7.4 |
| 68,961-92,185 | 24 | 5.8 |
| 9,226-27,650 | 109 | 26.1 |
| 92,186-1,84,375 | 55 | 13.2 |
| **Community** |  |  |
| General | 90 | 21.6 |
| OBC | 192 | 46.0 |
| Scheduled caste | 135 | 32.4 |
| **Marital Status** |  |  |
| Married | 378 | 90.6 |
| Single | 39 | 9.4 |
| Total | 417 | 100 |

**Table 2: Knowledge, Attitude, and Practice Regarding NOHP with 95% Confidence Intervals (N=417)**

| **Knowledge domain** | **Response** | **Frequency** | **Percentage** |
| --- | --- | --- | --- |
| Have you heard about/come across any program related to oral health? | No | 389 | 93.3 |
|  | Yes | 28 | 6.7 |
| Do you know about the services available under any program related to oral health being provided in your village? | No | 392 | 94 |
|  | Yes | 25 | 6 |
| Do you know about any oral health related camps been organised in your village? | No | 399 | 95.6 |
|  | Yes | 18 | 4.4 |
| Do you know about various oral diseases and its prevention? | No | 376 | 90.16 |
|  | Yes | 41 | 9.84 |
| Do you know about any oral health education session conducted in your village? | No | 401 | 96.16 |
|  | Yes | 16 | 3.84 |
| **Attitude domain** |  |  |  |
| **1.** Do you think oral health program ought to be implemented in the state? | No | 102 | 24.5 |
|  | Yes | 315 | 75.5 |
| **2.** Would you avail the dental services made available under the NOHP? | No | 64 | 15.3 |
|  | Yes | 353 | 84.7 |
| **3.** Would you recommend your near/dear ones to avail the dental services made available under the NOHP? | No | 42 | 10.1 |
|  | Yes | 375 | 89.9 |
| **4.** Do you think NOHP will reduce the burden of oral health diseases? | No | 40 | 9.6 |
|  | Yes | 377 | 90.4 |
| **5.** Do you think NOHP will lessen the expenses on oral health treatment? | No | 42 | 10.1 |
|  | Yes | 375 | 89.9 |
| **6.** Do you think setting up Dental Care Units at District and Sub-levels is a good initiative under NOHP ? | No | 44 | 10.6 |
|  | Yes | 373 | 89.4 |
| **7.** Do you think sensitization of the community about Basic Oral Health Care under NOHP is a welcome initiative? | No | 73 | 17.5 |
|  | Yes | 344 | 82.5 |
| **Practice domain** |  |  |  |
| Have you ever visited the nearest PHC/CHC for availing dental health services? | No | 407 | 97.6 |
|  | Yes | 10 | 2.4 |
| Are you visiting the Referral Centre for the follow-up visits? | No | 408 | 97.8 |
|  | Yes | 9 | 2.2 |
| Have you ever availed any dental treatment in the last 1 year? | No | 409 | 98.1 |
|  | Yes | 8 | 1.9 |
| Do you currently smoke any tobacco product, such as Bidis, Cigarettes, Cigars or Pipes? | No | 3 | .7 |
|  | Yes | 414 | 99.3 |
| Do you smoke tobacco products on a daily basis? | No | 22 | 5.3 |
|  | Yes | 22 | 5.3 |
| Were you young when you first started smoking? | No | 395 | 94.7 |
|  | Yes | 22 | 5.3 |
| During the past 12 months, have you ever tried to quit smoking? | No | 401 | 96.2 |
|  | Yes | 16 | 3.8 |
| During any visit to a doctor or other health worker in the past 12 months, were you counselled to quit smoking tobacco? | No | 412 | 98.8 |
|  | Yes | 5 | 1.2 |
